# Response of distribution patterns of two closely related species in *Taxus* genus to climate change since last inter‐glacial

**DOI:** 10.1002/ece3.9302

**Published:** 2022-09-14

**Authors:** Xingtong Wu, Minqiu Wang, Xinyu Li, Yadan Yan, Minjun Dai, Wanyu Xie, Xiaofen Zhou, Donglin Zhang, Yafeng Wen

**Affiliations:** ^1^ Central South University of Forestry and Technology Hunan China; ^2^ University of Georgia Athens Georgia USA

**Keywords:** Climate change, Elevational differences, Geographic distribution, Species distribution modeling, *Taxus chinensis*, *Taxus mairei*

## Abstract

Climate change affects the species spatio‐temporal distribution deeply. However, how climate affects the spatio‐temporal distribution pattern of related species on the large scale remains largely unclear. Here, we selected two closely related species in *Taxus* genus *Taxus chinensis* and *Taxus mairei* to explore their distribution pattern. Four environmental variables were employed to simulate the distribution patterns using the optimized Maxent model. The results showed that the highly suitable area of *T. chinensis* and *T. mairei* in current period was 1.616 × 10^5^ km^2^ and 3.093 × 10^5^ km^2^, respectively. The distribution area of *T. chinensis* was smaller than that of *T. mairei* in different periods. Comparison of different periods shown that the distribution area of the two species was almost in stasis from LIG to the future periods. Temperature and precipitation were the main climate factors that determined the potential distribution of the two species. The centroids of *T. chinensis* and *T. mairei* were in Sichuan and Hunan provinces in current period, respectively. In the future, the centroid migration direction of the two species would shift towards northeast. Our results revealed that the average elevation distribution of *T. chinensis* was higher than that of *T. mairei*. This study sheds new insights into the habitat preference and limiting environment factors of the two related species and provides a valuable reference for the conservation of these two threatened species.

## Graphical abstract

For *T. chinensis* and *T. mairei*, temperature and precipitation are the dominant factors limiting their distribution area. The distribution region of the two species was almost in stasis from LIG to the future periods. *T. chinensis* is mainly distributed in Sichuan Basin and surrounding mountains, and *T. mairei* occupies most of the mountain areas in eastern and central China.

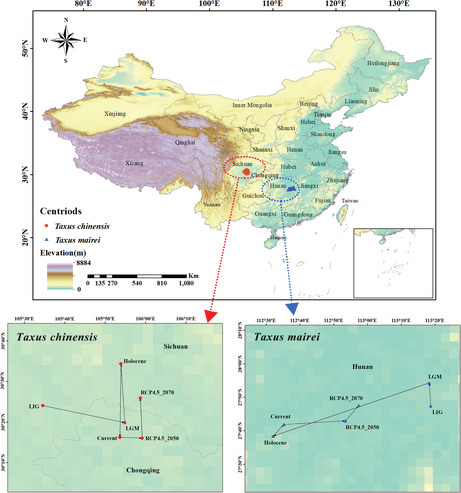

## 1 INTRODUCTION

Climate is a major factor that has effect on the habitat adaptability of most organisms worldwide. The quaternary period is characterized by distinct climatic oscillations. The average global temperatures and precipitation have fluctuated dramatically between glacial and inter‐glacial periods (Hou et al., 2020; Tsumura et al., 2020). Environmental alterations associated with climate change are altering the suitable habitats for many organisms, and species respond to these changes via migration and/or adaptation (Bystriakova et al., 2014; He et al., 2019; Liang et al., 2018). These migrations will produce new species combinations and species interactions (Guo et al., 2021; Liu et al., 2014; Wang et al., 2020). Meanwhile, migrations will pose a threat of local extinction for many species and/or accelerate the reproduction of some species (Dullinger et al., 2012; Elsen & Tingley, 2015; Faleiro et al., 2018; Hulme, 2017; Wiens, 2016; Zhang et al., 2019). Therefore, it is crucial to understand how climate change alters the distribution of species. Additionally, ample evidence has shown that land‐use changes (Fischer et al., 2008; Guo et al., 2019; Li et al., 2020; Madella et al., 2021; Ru, 2006), topography alterations (Elsen et al., 2020; Keppel et al., 2017; Wang et al., 2019), and human behaviors (Gallardo et al., 2015; Pecl et al., 2017) also play a non‐negligible role in regulating the effect of environmental changes on species distribution. It is essential to assess the species' habitat by adopting integrated variables and take effective measures to protect the ecological systems.

Species distribution models (SDMs) aim at predicting habitat suitability by integrating species distribution data and environmental data. SDMs have extensively been used to hind‐cast and predict future species distribution range change, to assess the impact of species invasion, to reveal niche conservatism, to provide guidance for species reintroduction site selection and conservation strategy formulation (Elith & Leathwick, 2009; Lenoir et al., 2009; Thuiller et al., 2019). Various approaches have been developed to construct SDMs such as Bioclim (Booth et al., 2014), Generalized Linear Models (Guisan et al., 2002), Random Forest (Breiman, 2001), and Maximum Entropy (MaxEnt) (Phillips et al., 2006). Here, we adopted the top‐performing Maximum Entropy (MaxEnt) approach which has been widely used in SDMs due to its simple clear graphical interface, high prediction accuracy, and its easy‐to‐understand output (Elith et al., 2011; Lissovsky & Dudov, 2021; Phillips et al., 2006; Phillips & Dudík, 2008). However, latest research has shown that MaxEnt is prone to over‐fitting, resulting in low model transfer ability, which seriously affects its application to various fields such as the invasion biology and phylogeography (Jiménez‐Valverde et al., 2011; Syfert et al., 2013; Zhu & Qiao, 2016). Model complexity of MaxEnt is mainly affected by the 4 parameters, namely, background data, feature class (FC), regularization multiplier (RM), and sampling bias (Merow et al., 2013). Recently, Muscarella et al. (2014) have developed the ENMeval package to perform automated tuning and evaluations of species distribution models. This species‐specific tuning in Maxent settings can avoid over‐fitting in niche models and improve predictive ability.

*Taxus* is the largest and most widely distributed species in Taxaceae (Fu et al., 1999). In the 1980s, *Taxus* attracted great attention, meanwhile suffering huge damage since the Taxol extracted from it was found to be one of the most popular natural anticancer materials (Li et al., 2020). Due to over‐exploitation and anthropogenic disturbances, *Taxus* population diminished sharply and become fragmented. At present, it is at a high risk of extinction (Liu et al., 2011, 2018; Yu et al., 2014). Moreover, its biological properties such as low pollination rate, long seed dormancy, and weak competitive ability of seedlings also cause its current endangered status (Li et al., 2015; Liu, Feng, et al., 2019; Liu, Wang, et al., 2019). Internationally, the three *Taxus* species (*Taxus wallichiana*, *Taxus contorta*, and *Taxus chinensis*) are listed as endangered species (EN, Thomas et al., 2013; Thomas, 2011; Thomas & Farjon, 2011), and *Taxus mairei* as Vulnerable (VU, Yang et al., 2013), and *Taxus cuspidata* as the least concern (LC, Katsuki & Luscombe, 2013) on the IUCN Redlist. In China, *Taxus* species have been listed as a national first‐class protected plant (State Forestry Administration of China, 1999). It is worth mentioning that the two closely related *Taxus* species *Taxus chinensis* and *Taxus mairei* are mainly distributed in the Sino‐Himalayan forest floristic subkingdom and the Sino‐Japanese forest floristic subkingdom (Wu & Wu, 1996). *Taxus chinensis* is endemic to China, and it is mainly distributed in the mountains around Sichuan Basin. *Taxus mairei* has a wide distribution in the south of the Yangtze River in China and other countries of South Asia and Southeast Asia. Generally speaking, *Taxus mairei* is usually at lower elevation than *Taxus chinensis* (Fu et al., 1999). However, the morphological characteristics of the two species are almost the same, and thus it is difficult to distinguish them (Farjon, 2017). As an ancient and long‐lived tree species, the two species experience Quaternary glaciation (Liu et al., 2018; Möller et al., 2020). However, how the two species respond to elevation change and climatic oscillation in the Quaternary remains to be further explored (Wang et al., 2019). With the increasing energy consumption, global warming is one of the main challenges in the 21st century (Durán‐Martín et al., 2019). The distribution change of these two species in response to global warming is largely unknown. Hence, the mappings of suitable habitats and predictions of the impacts of climate change are vital for habitat protection and the sustainable development of these two species.

In this study, we integrated optimized species distribution models (SDMs) and geographical information system (GIS) software to analyze the two species distribution pattern in response to climate change with the aims to (1) uncover the dominant environmental factors in their niche differentiation; (2) determine elevation differences of the two species since last inter‐glacial (LIG) periods; (3) reveal the conservation implications for the species. Overall, this study will deepen our understanding of their evolutionary history and provide some useful guidelines for the conservation of these two threatened species.

## 2 METHODS

### 2.1 Species occurrence data

**FIGURE 1:**
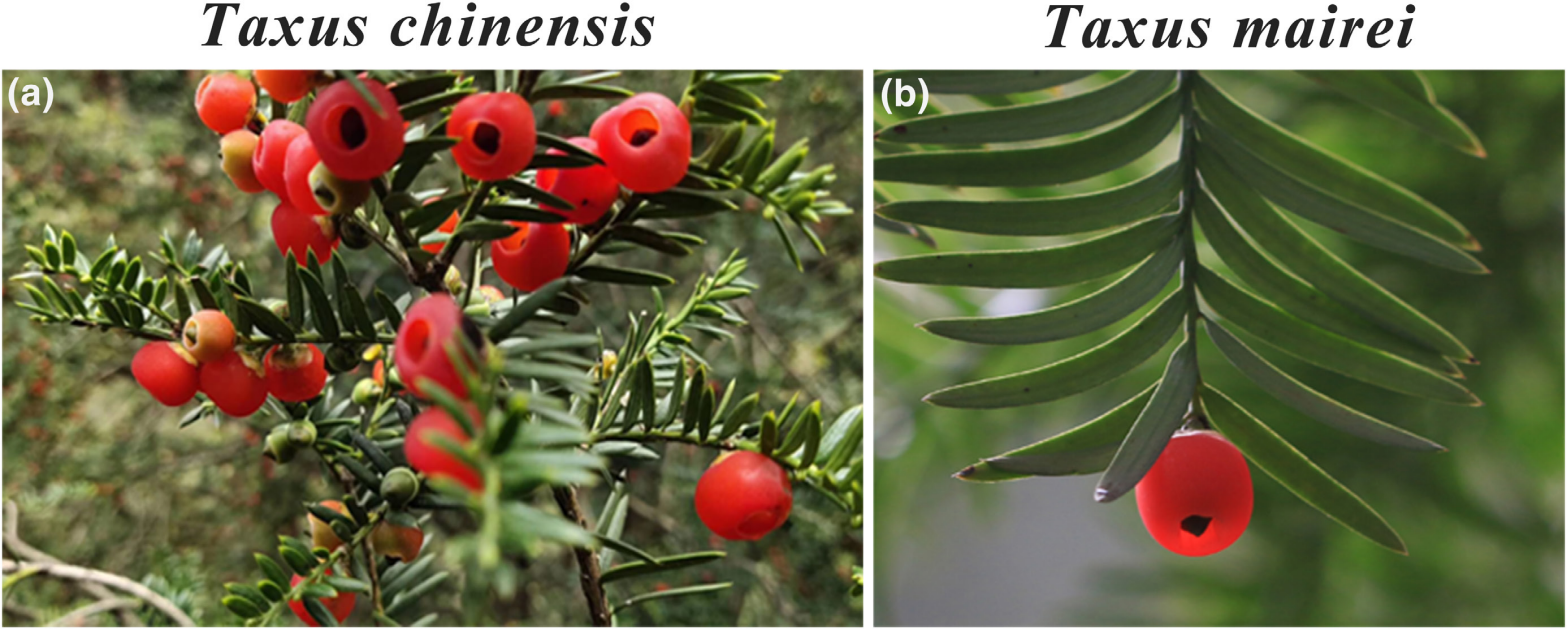
Organism photograph of the two species. (a) *Taxus chinensis* from Zhen'an, Shaanxi. Leaves linear, straight to distally falcate, usually (0.8‐) 1.5–2.2 cm × 2–3.2 mm. (b) *Taxus mairei* in Qiyang, Hunan. Leaves linear, usually falcate and 1.5–3.5 cm × 2–4 mm.

Organism photographs in the fruiting stage (August–December) of *T. chinensis* and *T. mairei* were shown in Figure 1. Species occurrence data of *T. chinensis* and *T. mairei* were collected from the fieldwork, previous studies (Liu et al., 2011, 2018), the Chinese Virtual Herbarium (CVH, http://www.cvh.ac.cn/) and the Plant Photo Bank of China (PPBC, http://www.plantphoto.cn/). (Number of occurrence data for each species from each dataset was shown in Table S1.) Due to *Taxus chinensis* and *Taxus mairei* were mainly distributed in China, and the aim of the study is to infer the potential distribution area of the two species in China; thus, we do not consider the Global Biodiversity Information Facility (GBIF) database in the current study. Then, the data with obvious geographical coordinates errors were removed by the ArcGIS 10.4. In addition, the duplicate data were removed to ensure only one record in the 2.5′ × 2.5′ grid by the “spThin” package. Finally, a total of 63 sampling points for *T. chinensis*, and 140 sampling points for *T. mairei* were retained (Figure S1; Table S1).

### 2.2 Predictor variables

The relevance and completeness of variables are key components for constructing SDMs (Elith & Leathwick, 2009; Guo et al., 2017; Zimmermann et al., 2010). Four types of environmental variables (bioclimatic, topographical, soil variables, and human interference index) were selected. Nineteen bioclimatic variables were downloaded from WorldClim 2.1 (https://worldclim.org) for the current period (1970–2000) at a 2.5 arc minutes spatial resolution. To avoid biased estimates of model coefficients and spurious significance levels resulting from multi‐collinearity, we excluded highly correlated climate variables based on Pearson's correlation coefficient (|r| > 0.70) and retained 6 climatic variables for each of the two species (Table 1; Figure S2). Three topographical variables, including the elevation obtained from the WorldClim database (https://worldclim.org), and the slope and aspect obtained from Digital Elevation Model using the 3D analyst tools in the software ArcGIS 10.4. Five soil variables were downloaded from the Harmonized World Soil Database (HWSD, https://www.fao.org/soils‐portal/data‐hub/soil‐maps‐and‐databases/harmonized‐world‐soil‐database‐v12/en/) based on previous studies (Guo et al., 2019; Li, Zhang, & Griffith, 2021; Li, Zhang, Zhu, et al., 2021; Ru, 2006), and the correlation coefficient between the five soil variables was less than 0.7 (Figure S3). Human interference index (HII) was downloaded from Socioeconomic Data and Applications Center (SEDAC, http://sedac.ciesin.columbia.edu) (Gallardo et al., 2015; Madella et al., 2021). Ultimately, 15 ecological variables were chosen for each species for further analysis (Table 1).

**TABLE 1:** Contribution rate and importance of environmental variables for *Taxus chinensis* and *Taxus mairei*

Type	Variables	Taxus chinensis		Variables	Taxus mairei	
		Percent of Contribution (%)	Permutation importance (%)		Percent of Contribution (%)	Permutation importance (%)
Climate	bio2	28.4	5.7	bio4	2.0	13.4
	bio5	10.8	0.2	bio5	0.5	4.3
	bio7	9.7	2.4	bio8	3.1	3.2
	bio11	39.4	58.4	bio11	24.1	16.5
	bio13	0.2	1.8	bio15	9.2	14.2
	bio15	0.1	0.1	bio18	34.7	16.9
Topographical	Elevation	5.4	17.0	Elevation	14.2	8.0
	Aspect	0.1	0.1	Aspect	0.6	1.7
	Slope	0.6	0	Slope	3.8	3.4
Soil	Nutrient availability	2.3	12.0	Nutrient availability	0	0
	Rooting conditions	0.4	0.5	Rooting conditions	0.3	15.6
	Oxygen availability to roots	0.8	0.8	Oxygen availability to roots	0	0
	Topsoil Base Saturation	0.5	0.3	Topsoil Base Saturation	6.4	0.9
	Available water storage capacity	0.1	0	Available water storage capacity	0.3	0.9
Human influence	Human influence index	1.3	0.8	Human influence index	0.8	0.8

*Note*: bio2: Mean Diurnal Range; bio4: Temperature Seasonality; bio5: Max Temperature of Warmest Month; bio7: Temperature Annual Range; bio8: Mean Temperature of Wettest Quarter; bio11: Mean Temperature of Coldest Quarter; bio13: Precipitation of Wettest Month; bio15: Precipitation Seasonality; bio18: Precipitation of Warmest Quarter.

Furthermore, bioclimatic variables of Last inter‐glacial (LIG, 120,000–140,000 years BP), Last Glacial Maximum (LGM, About 22,000 years ago, CCSM model), and Mid Holocene (About 6000 years ago, CCSM model) were obtained from CMIP 5. Future bioclimatic variables for the 2050 (2041–2060) and 2070 (2061–2080) were obtained from RCP4.5 (Representative Concentration Pathways) based on the Community Climate System Model (CCSM). CCSM model is a coupled climate model for simulating the earth's climate system, whereas RCP4.5 provides a platform for climate models to explore the climate system response to stabilizing the anthropogenic components of radiative forcing (Thomson et al., 2011). However, there are no available data on the other three types of environmental variables (topographical variable, soil variables, and human interference index) for the past and future periods corresponding to the same periods. Since the two species are mainly distributed in the mountain areas, they are relatively less influenced by topograghy, soil, and human behaviors. Therefore, these three types of environment variables were assumed to be constant, as reported in previous studies (Evans et al., 2020; Lv et al., 2021; Zhang et al., 2019).

### 2.3 Modeling procedures

We conservatively defined the background range as an area surrounding our occurrence location by buffering a bounding box. We set the buffer distance as 7 degrees (about 779 km) based on the dispersion distance of the *Taxus* pollens and seeds (Brown et al., 2017; Li et al., 2015; Li, Zhang, & Griffith, 2021; Li, Zhang, Zhu, et al., 2021). A total of 10,000 background points were randomly generated using the function randomPoints in the dismo package (Hijmans et al., 2021). ENMeval package was employed to assess model performance by tuning the combination between regularization multiplier (RM) and feature classes (FC) (Kass et al., 2021; Muscarella et al., 2014). The regularization multiplier (RM) range was set as 0.5 to 5.0, increasing by 0.5 each time; and six feature classes (FC) included L, H, LQ, LQH, LQHP, and LQHPT (L = linear, Q = quadratic, H = hinge, P = product, and T = threshold) (Kass et al., 2021; Lissovsky & Dudov, 2021). The model with the lowest Delta Akaike information criterion (Delta‐AICc = 0) value was selected (Akaike, 1998). According to the relevant parameter ahead, Maxent v3.4.1 was used to investigate the effects of past and present climatic conditions on *T. chinensis* and *T. mairei* (Phillips et al., 2006). The 75% of species occurrence data and the remaining 25% were used as training data and testing data for model validation, respectively. The 10 replications and 5000 bootstrap iterations were set, and other parameters had the default settings. Model performance was evaluated by the area under the receiver operating characteristics curve (AUC). The AUC ranged from 0 to 1. The model with AUC value of more than 0.9 was considered as excellent (Araújo et al., 2005; Elith et al., 2006). To determine the environmental variables with the greatest effect for the model, the percentage contribution and the permutation importance were investigated. The contribution of each variable to the regularized gain of the model was quantified as percentage contribution. The values in the corresponding column in the input matrix were permuted, and MaxEnt's gain before and after permutation was compared to obtain each variable permutation importance (Almeida et al., 2022; Valencia‐Rodríguez et al., 2021). The continuous distribution probability was classified by using the “reclassify” function in ArcGIS 10.4. The study area was classified as either a unsuitable area (*p* < 0.1) and suitable area (0.1 ≤ *p* < 1.0), and the suitable area was sub‐classified as a poorly suitable area (0.1 ≤ *p* < 0.3), moderately suitable area (0.3 ≤ *p* < 0.5), and highly suitable area (0.5 ≤ *p* < 1.0) (Cao et al., 2016; Liu et al., 2021).

### 2.4 Distribution area change and centroid transition

Based on the results of the species distribution modeling, SDMtoolbox (Brown et al., 2017) in ArcGIS v10.4 was used to evaluate the changes in the area during different periods and the centroid shift for the two *Taxus* species. We cross‐checked the changes of the highly suitable area to identify regions as (i) expansion, (ii) unchanged, and (iii) contraction relative to the previous periods (Hu et al., 2019). Centroid shift concentrated the species distribution on an independent central point and created a vector file to depict the magnitude and direction of changes over time (Cong et al., 2020; Hu et al., 2015).

## 3 RESULTS

### 3.1 Model accuracy and contributions of predictor variables

Species distribution modeling was constructed for each species to predict its geographical distributions at present, in the past and future. Based on the results of ENMeval, the optimal combination (Delta‐AICc = 0) (Figure S4) of RM/FC for *T. chinensis* and *T. mairei* was 0.5/LQ and 1/LQH, respectively. These parameters combination could avoid model's over‐fitting and improve its prediction ability. Our data showed that the area under the receiver operating characteristics curve (AUC) obtained from all the models was larger than 0.9 (AUC > 0.9), indicating the robustness and reliability of predictions of our models (Figure S5).

The contribution of each predictor variable suggested that the two species had different environmental requirements. Mean temperature of coldest quarter (bio11), mean diurnal range (bio2), and max temperature of warmest month (bio5) were the three most important predictor variables determining the distribution of *T. chinensis*, while precipitation in warmest quarter (bio18), the mean temperature in coldest quarter (bio11), and elevation were the determining factors for the potential distribution of *T. mairei* (Table 1). For *T. chinensis*, the top three permutation importance factors mean temperature of coldest quarter (bio11), elevation, and nutrient availability accounted for 87.4%. For *T. mairei*, the sum of precipitation in warmest quarter (bio18), Mean temperature of coldest quarter (bio11), and rooting conditions reached 49.0% (Table 1). The analysis results of percent contribution and permutation importance indicated that temperature and precipitation were the most important environment factors for the distribution patterns of two species.

**FIGURE 2:**
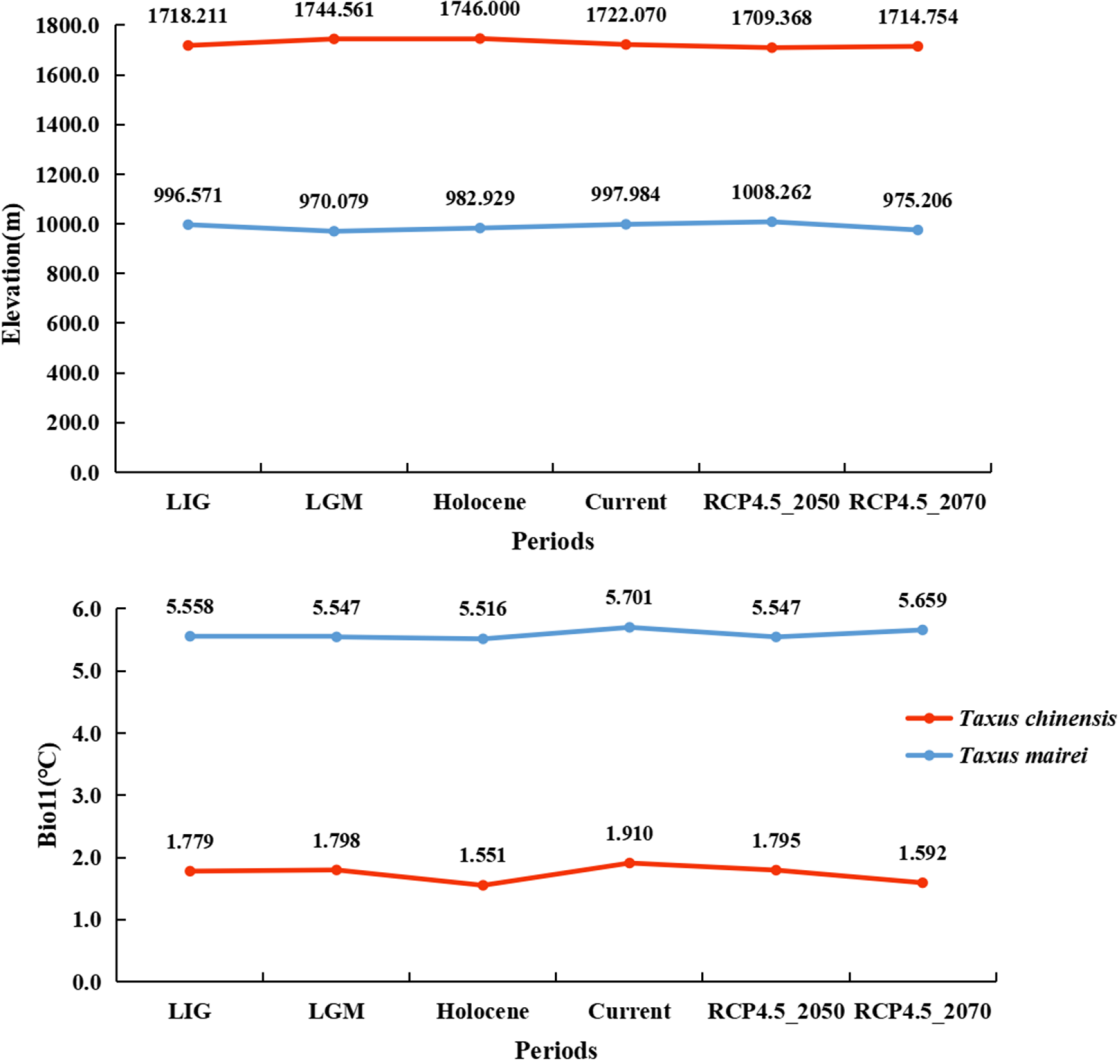
Changing tendency of elevation and bio11 under different climate periods

Bio11 was the most percentage contribution factors for the distribution of *T. chinensis* while it was the second contribution factors for *T. mairei*. The kernel density of the occurrence probability of the two species versus the mean temperature of coldest quarter (bio11) were presented in Figure S6. The kernel density clearly showed that the two *Taxus* species had different temperature preferences, and the suitable temperature was about −10 to 15°C for *T. chinensis* and −5 to 20°C for *T. mairei* with an average temperature of 1.79 and 5.60°C, respectively (Figure 2; Figure S6).

### 3.2 Distribution area during different periods

**FIGURE 3:**
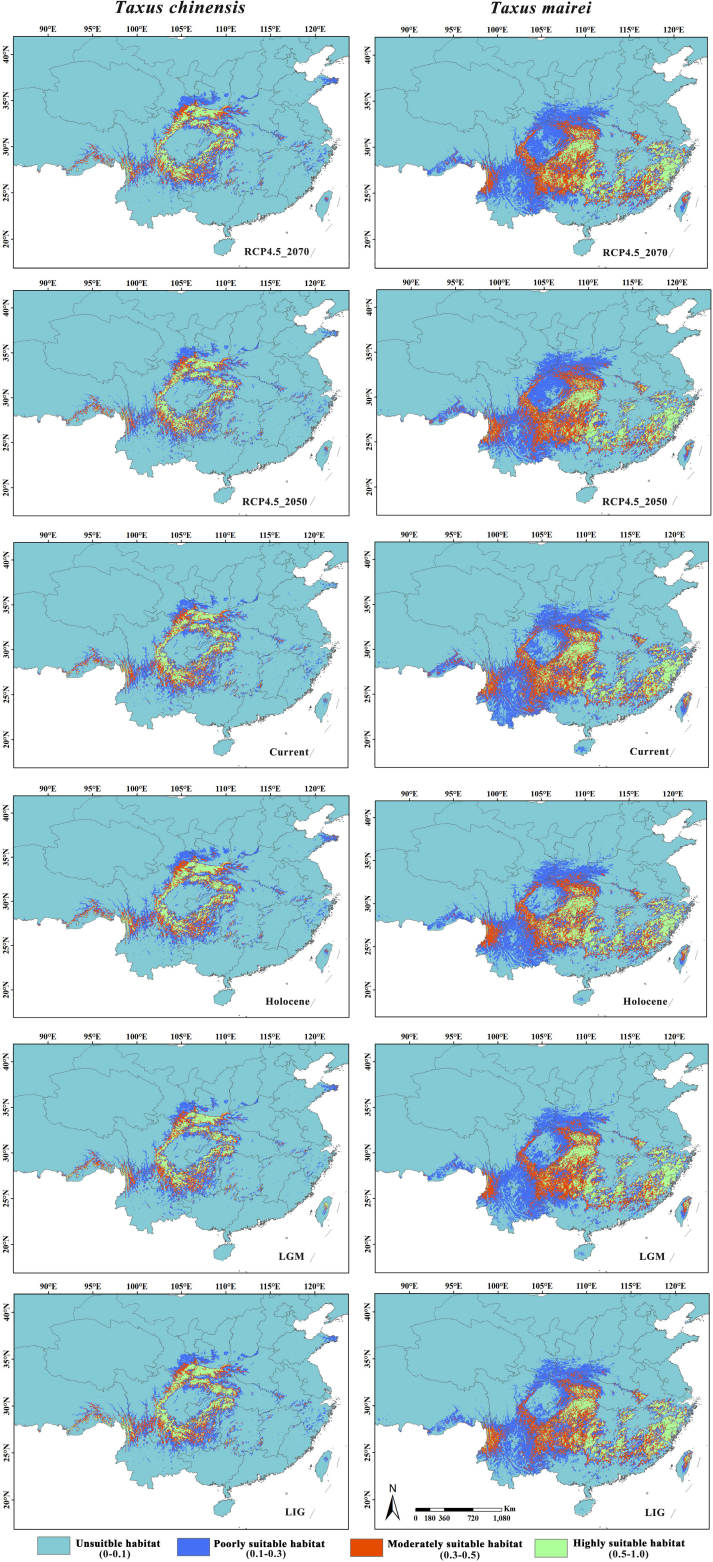
Potential distribution of the *Taxus chinensis* and *Taxus mairei* under different climate periods

The potential distributions of the two species during different climatic periods were presented in Figure 3. The suitable area (0.1 ≤ *p* < 1.0) of the two species was partially overlapped, but the highly suitable area (0.5 ≤ *p* < 1.0) was scarcely overlapped. *T. chinensis* was mainly distributed around the Sichuan Basin, while *T. mairei* occupied most of regions in the eastern and central parts of China.

**FIGURE 4:**
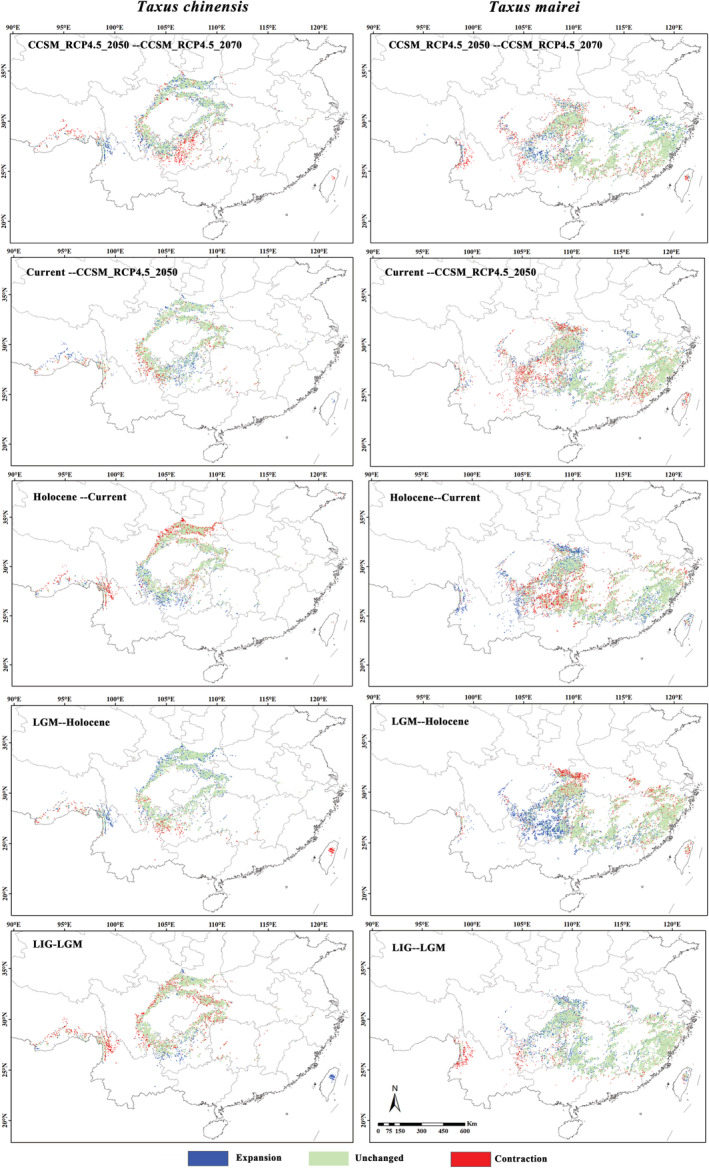
Changes in distribution area of the *Taxus chinensis* and *Taxus mairei* between two adjacent periods

From the Last inter‐glacial (LIG) to the future periods (RCP4.5_2070), the highly suitable area (0.5 ≤ *p* < 1.0) of *T. chinensis* shown an overall trend of shrinking (−3.95%) while *T. mairei* shown the general tendency of expansion (7.07%) (Table 2; Figure 4). The highly suitable area of *T. chinensis* was smaller than that of *T. mairei* in different periods (Table 2). The highly suitable area of *T. chinensis* was 1.616 × 10^5^ km^2^ in current period, and it was decreased by 8.86% compared with that in LIG period. However, from current to the future (RCP4.5_2070), its area was increased by 5.38%. The average loss and gain percentage was 14.22% and 13.00%, respectively. The highly suitable area of *T. mairei* was 3.093 × 10^5^ km^2^ in current period, which was 11.54% higher than that in the LIG period. Nevertheless, it shrank by 4.01% from current period to the future period. The average loss and gain percentage was 12.43% and 13.80%, respectively (Table 2).

**TABLE 2:** Changes in the distribution area of *Taxus chinensis* and *Taxus mairei* in different periods and different scenarios

Species	Periods	Suitable area (×10^5^ km^2^) (%)	Highly suitable area (×10^5^ km^2^) (%)	Contraction (×10^5^ km^2^)	Stable (×10^5^ km^2^)	Expansion (×10^5^ km^2^)	Percentage loss(%)	Percentage gain (%)
*Taxus chinensis*	RCP4.5_2070	6.952 (−0.90)	1.703 (2.16)	0.244	1.517	0.280	14.32	16.44
	RCP4.5_2050	7.015 (−1.52)	1.668 (3.22)	0.165	1.539	0.221	9.89	13.24
	Current	7.123 (−7.21)	1.616 (−5.99)	0.299	1.507	0.197	18.50	12.19
	Holocene	7.677 (13.00)	1.719 (8.80)	0.128	1.544	0.261	7.45	15.18
	LGM	6.792 (−7.89)	1.580 (−10.89)	0.341	1.547	0.125	20.94	7.91
	LIG	7.374	1.773					
*Taxus mairei*	RCP4.5_2070	14.392 (−0.06)	2.969 (0.75)	0.352	2.831	0.373	11.86	12.56
	RCP4.5_2050	14.401 (−3.17)	2.947 (−4.72)	0.472	2.864	0.320	16.02	10.86
	Current	14.873 (3.83)	3.093 (−0.55)	0.490	2.878	0.459	15.84	14.84
	Holocene	14.325 (2.50)	3.110 (7.13)	0.36	2.777	0.590	11.58	18.97
	LGM	13.976 (0.89)	2.903 (4.69)	0.199	2.795	0.342	6.86	11.78
	LIG	13.854	2.773					

*Note*: Future: RCP4.5_2070 and RCP4.5_2050 under CCSM; Holocene: Holocene; LGM: Last Glacial Maximum; LIG: Last Inter Glacial. Changing of the distribution area were based on the previous periods. Suitable area, 0.1 ≤ *p* < 1; Highly suitable area, 0.5 ≤ *p* < 1.0.

### 3.3 Centroid migration

**FIGURE 5:**
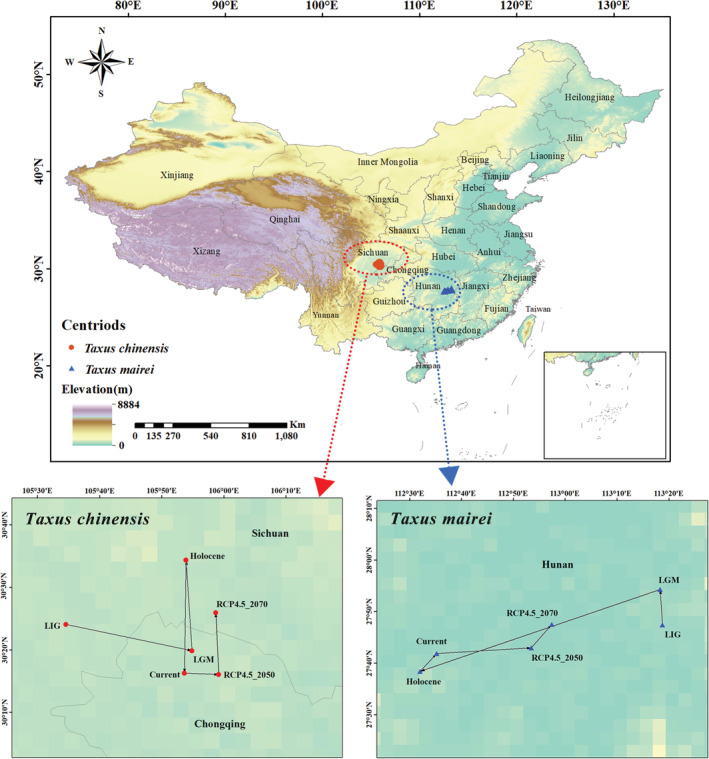
Centroid migration routes under different climate periods for *Taxus chinensis* and *Taxus mairei*

In different periods, the centroid of *T. chinensis* was distributed in the adjacent areas of Sichuan province and Chongqing city, while *T. mairei* was distributed in Hunan province (Figure 5). During the LIG period, the centroid of *T. chinensis* and *T. mairei* was located at 105.576E/30.401 N and 113.312E/27.790 N, respectively. Besides, the centroid of *T. chinensis* and *T. mairei* was located at 105.894E/30.271 N and 112.586E/27.697 N in current period. In the RCP4.5_2070, the centroid of *T. chinensis* and *T. mairei* was located at 105.978E/30.433 N and 112.955E/27.792 N, respectively (Table 3). From LIG to the current periods, the centroid of *T. chinensis* exhibited a migration trend towards southeast (33.880 km) and that of *T. mairei* towards southwest (70.844 km), respectively. From the current period to the future period (RCP4.5_2070), *T. chinensis* and *T. mairei* displayed a migration trend towards northeast, and its migration distance was 19.616 km and 37.242 km, respectively (Figure 5).

**TABLE 3:** Coordinates and the distance of the centroids

Periods	Taxus chinensis			Taxus mairei		
	Longitude (E)	Latitude (N)	Migration distance (km)	Longitude (E)	Latitude (N)	Migration distance (km)
RCP4.5_2070	105.978	30.433	18.26	112.955	27.792	10.67
RCP4.5_2050	105.986	30.268	8.86	112.89	27.715	29.44
Current	105.894	30.271	33.35	112.586	27.697	8.33
Holocene	105.899	30.573	26.72	112.532	27.639	80.27
LGM	105.914	30.331	33.52	113.304	27.905	13.00
LIG	105.576	30.401		113.312	27.790	

*Note*: Future: RCP4.5_2070 and RCP4.5_2050 under CCSM; LGM: Last Glacial Maximum; LIG: Last Inter Glacial. Migration of the distance were based on the previous periods.

The migration distance of each species between the two adjacent periods was shown in Table 3. For *T. chinensis*, the largest migration distance was 33.53 km from the LIG to the LGM periods. For *T. mairei*, the largest migration distance was from the LGM to the Holocene periods with the migration distance of 80.27 km.

### 3.4 Elevational differences

To reveal the elevation difference between the two species, we calculated the average elevation of the two species during different periods. The suitable elevation range for the two species was within 0–4000 m based on the kernel density. But the peak of the kernel density was different, it was about 1500 m and 800 m for *T. chinensis* and *T. mairei*, respectively (Figure S6). Furthermore, average elevation of the occurrence data indicated that the elevation of *T. chinensis* (1715.5 m) was higher than that of *T. mairei* (985.8 m) (Figure 2).

From the LIG to the current period, the elevation of *T. chinensis* and *T. mairei* increased by about 4 and 1 m, respectively. In the future period (RCP4.5_2070), the general trend of the elevation of *T. chinensis* and *T. mairei* is downward with the increase in temperature (Figure 2).

## 4 DISCUSSION

### 4.1 Main factors affecting distribution of two *Taxus* species

*T. chinensis* and *T. mairei* are two species widely distributed in the subtropical and warm temperate zones in China. The biological characteristics of the two species and phenological observations indicate that *Taxus* is shade‐tolerant species, and that it prefers to grow along the river (Liu et al., 2013; Song, 2013). In this study, the mean temperature of the coldest quarter (bio11) was found to be the most important and most contributing factor for *T. chinensis*. Thus the temperature was the main factor influencing its spatial distribution. For *T. mairei*, the most contribution and permutation importance factor was bio18 (precipitation in warmest quarter). Wang et al. (2019) have shown that annual precipitation (bio12) and topographical variables have a strong effect on the distribution of *T. chinensis* and *T. mairei*. Li et al. (2022), Liu, Feng, et al. (2019) and Liu, Wang, et al. (2019) have also supported that precipitation is the most important climatic factor that restricts the habitat distribution of the *T. mairei*. Poudel et al. (2012) have reported that great differences in rainfall between winter (low) and summer (high) are the determining factor responsible for the distribution of *T. mairei* in the east of the Himalayas in Nepal. Overall, precipitation is the dominant factor determining the distribution of the *T. mairei*.

It is worth mentioning that rooting condition is the third permutation importance factor (15.6%) for *T. mairei* (Table 1). Ru (2006) has shown that *T. mairei* prefers living in an environment with moist fertile soil and good water permeability. Owing to *T. mairei* lives in relatively low elevation areas with ample environment moisture; therefore, good water permeability is conducive to the growth of *T. mairei*.

### 4.2 Changes in species distribution area

The distribution area of *T. chinensis* and *T. mairei* were almost in stasis from the LIG to the future (RCP4.5_2070). This can be understood from the following two points. First, biological traits such as limited dispersal capacity, long generation time and low rate of seed germination of *Taxus* (Keppel et al., 2017; Li, Zhang, & Griffith, 2021; Li, Zhang, Zhu, et al., 2021; Ru, 2006; Wang et al., 2018, 2019). Second, the main distribution region of the natural populations of the two species were in the mountains such as the Qinling, Nanling, and Wuyi Mountains in China (Fu et al., 1999; Li et al., 2022). *T. chinensis* is mainly distributed around the Sichuan Basin, while *T. mairei* occupies most of the southern regions of the Qinling‐Daba Mountains in China. These mountains not only provide a relatively stable habitat for species but also act as the refuge (Jiang et al., 2019; Keppel et al., 2017; Ye et al., 2017; Zhao et al., 2019). This phenomenon has also been reported on *T. mairei*, *T. wallichiana* (Wang et al., 2019), *Tsoongiodendron odorum* (Hu et al., 2020), and *Houttuynia cordata* (Liu et al., 2021) and *Eucalyptus grandis* (Ouyang et al., 2022). Species distribution models (SDMs) are based on the niche conservatism hypothesis and niche conservatism is more prevalent than niche differentiation (Alexander, 2013; Chivers et al., 2017). We have adjusted parameters many times to test the changing tendency of the species distribution area between two adjacent periods, the end result, however, was about the same. Thus, the models was not the main reasons that lead to the overall stasis in the species distribution area of the two species.

Notably, the suitable area of the two species will shrink from current period to future period because the plant growth, development and reproduction are vulnerable to the effects of increasing global temperature (Liu, Feng, et al., 2019; Liu, Wang, et al., 2019). Our result is consistent with the previous findings of study of *T. mairei* (Li, Zhang, & Griffith, 2021; Li, Zhang, Zhu, et al., 2021). Meanwhile, the suitable area of *Taxus cuspidata* and *Taxus wallichiana* will be reduced with the rising temperature in the future (the 2050 and 2070) (Li et al., 2020; Su et al., 2018). This shrinking tendency is also observed in other species such as *Quercus lamellosa* (Guo et al., 2021) and *Polyporus umbellatus* (Guo et al., 2019).

Species may change its latitude or elevation in response to climate changes (Davis & Shaw, 2001). Previous studies have shown that species will move northward and upwards with the increasing temperature, such as *Quercus lamellosa* (Guo et al., 2021), *Cyananthus* (He et al., 2019), *Quercus kerrii* (Jiang et al., 2018), wild soybean (He et al., 2016), and *T. wallichiana* (Li et al., 2020). Likewise, this study also found an upward and northward shift trend for *T. mairei*, which is consistent with the reports by Li, Zhang, and Griffith (2021), Li, Zhang, Zhu, et al. (2021) and Poudel et al. (2012). Instead, *T. chinensis* shifted downward and northward in China. This may be due to the fact that survival pressure from the higher elevation is not conducive to the growth and reproduction of *T. chinensis*, and that the special topography around the Sichuan Basin may provide a route for *T. chinensis* to migrate northward. Furthermore, Liang et al. (2018) modeled 151 representative plants in the Hengduan Mountains and its adjacent areas in China, and found that the mountain plants shifted upward with the increasing temperature, but the shift was not only northward but also westward or in other directions.

### 4.3 Elevational differences of two *Taxus* species

Elevational differences were observed between the two closely related *Pinus* species *Pinus massoniana* and *Pinus hwangshanensis*. The reason for such elevational differences lie in that species specificity and climatic divergence selection of the candidate genes play a key role in the ecological divergence of these two species (Li et al., 2010; Zhou et al., 2014). Theoretically, closely related species are expected to show more similarity as a consequence of shared climate selection, habitat, and evolutionary history (Miller et al., 2019; Nürk et al., 2015). Recent climatic selection may be species‐specific since forest trees typically have the highest adaptation in their own environment (local adaptation), and different species typically occupy different climatic niches (ecological niche differences) (Hua & Wiens, 2013; Savolainen et al., 2007). Our species distribution models (SDMs) results indicated that *T. chinensis* is mainly located at the elevation above 1500 m, and *T. mairei* tends to occur at the elevation of around 1000 m since LIG. Our results are in accordance with the description of *Flora of China* (Fu et al., 1999). Species distribution models results shown that there is no large‐scale population migration for the two closely related species. Temperature and precipitation are the main factors determining species distribution regions. Therefore, this study shown that climatic selection and long‐term adaption to a given environment might the main factors that influencing the two species divergence along the elevation. Furthermore, we have observed a hybrid zone (*T. chinensis* and *T. mairei*) in the intermediate transition zone between high altitude and low altitude region (unpublished data) in the nature reserve, and this hybrid zone provides good materials for us to explore the dynamic history of the two closely related species at the molecular level.

### 4.4 Implications for conservation and management

From current to future periods (RCP4.5_2070), the suitable distribution area of *T. chinensis* and *T. mairei* will shrink. First, upward migration of species along elevational gradients will lead to range contraction for many species since the total area available at a given altitude usually decreases with increasing elevation in mountains (Parmesan, 2006; Wilson et al., 2005). Second, from the view of biological characteristics, *Taxus* prefers a shady and humid environment (Wu & Wen, 2017). However, with the rising temperature, the climatic conditions such as strong radiation, drought, wind, and other adverse climatic conditions will be more severe in high‐altitude areas (Ouyang et al., 2022; Solomon et al., 2007; Yin et al., 2020). Thus, these adverse conditions will pose relatively more physiological constraints on *T. chinensis*, thus resulting in range reduction.

However, the reason for the range contraction of *T. mairei* may be different from that of *T. chinensis*. Rooting conditions is the third most important factor that affects the distribution of *T. mairei*. Besides, the increasing studies have shown that temperature and precipitation are the main factors that affect rooting (Fang et al., 2017; Reich et al., 2018). In general, global warming is expected to cause changes in distribution, intensity, and frequency of precipitation (Myhre et al., 2018). Inappropriate hydrothermal conditions are not conducive to the rooting of seeds, eventually resulting in the population number decline. Moreover, the anthropogenic disturbance is stronger in the low‐elevation areas than in high‐elevation areas, and hence it may also lead to the contraction in species' range.

Taken together, we can establish germplasm resource nurseries to cultivate the seeds from different provenances, especially for the *T. chinensis* distributed in high‐elevation areas. Furthermore, considering the influence of humans, in‐situ protection should be enforced for the samples that are easily accessible. Theoretically, evolution can also drive species distribution range shifts in the absence of environmental change (Holt, 2003; Parmesan, 2006), such as the inter‐species interactions, hybridization, and introgression, and thus common garden experiments should be undertaken to investigate potential local adaptations and facilitate the development of future genetic studies.

It should be noted that although predictions based on species distribution models (SDMs) effectively uncover the dynamic population history of the two species, there are also some limitations in current study. First, soil variables and human interference index are assumed to be constant in the ancient climate and future climate. However, species could be threatened, or even possibly become extinct in the case of a dramatic increase in human population and land use, thus resulting in habitat loss and fragmentation (Giam et al., 2010). Thus, appropriate protection measures should be taken when anthropogenic disturbances drastically increase. Additionally, the effects of solar radiation on the SDMs results are not taken into consideration in the current study (Ouyang et al., 2022). Because the two species are shade‐preferring species and were always the associated tree species, solar radiation may not have a direct impact on them (Liu et al., 2013; Su et al., 2018). However, we suggest that solar radiation should be taken into account in the future research, especially for the heliophilous species and those species distributed in the regions with high solar radiation such as Qinghai‐Tibet Plateau. Finally, for woody plants with long generations, the change of climate from suitable to unsuitable does not mean the disappearance of species distribution in a specified area, instead, unsuitable climate may involve more environmental stress that species may need to suffer. Thus, we should consider the effect of climate on species distribution pattern when evaluating the endangered category of the species.

## 5 CONCLUSION

Our findings enhance our understanding of the past and present plant species dynamics driven by climate change across the Sino‐Japanese floristic regions in China. For *T. chinensis* and *T. mairei*, temperature and precipitation are the dominant factors limiting their distribution area. The distribution region of the two species exhibited overall stasis from LIG to the future periods. *T. chinensis* is mainly distributed in Sichuan Basin and surrounding mountains, and *T. mairei* occupies most of the mountain areas in eastern and central China. Therefore, the Sichuan Basin region may be the key study area of their hybrid zones. In addition, long‐term adaption to the environment may be mainly responsible for the higher average elevation of *T. chinensis* than *T. mairei*. Furthermore, in response to climate warming, the suitable distribution area of the two species will shrink, and they were expected to move northwards. It should be noted that we should pay special attention to the potential habitat changes induced by climate change and focus on the impact of habitat on rooting conditions in *Taxus* conservation work.

## AUTHOR CONTRIBUTIONS

**Xingtong Wu:** Conceptualization (equal); data curation (lead); formal analysis (lead); investigation (lead); methodology (lead); software (lead); visualization (lead); writing – original draft (lead); writing – review and editing (lead). **Minqiu Wang:** Formal analysis (equal); investigation (equal); supervision (equal); writing – review and editing (equal). **Xinyu Li:** Formal analysis (equal); software (equal); supervision (equal); writing – review and editing (equal). **Yadan Yan:** Investigation (equal); validation (equal). **Minjun Dai:** Software (equal); visualization (equal). **Wanyu Xie:** Software (equal); supervision (equal). **Xiaofen Zhou:** Software (equal); supervision (equal). **Donglin Zhang:** Conceptualization (equal); supervision (equal); validation (equal); writing – review and editing (equal). **Yafeng Wen:** Conceptualization (equal); funding acquisition (lead); investigation (equal); resources (equal); supervision (equal); writing – review and editing (equal).

## CONFLICT OF INTEREST

The authors declare no competing interests.

## Supporting information


**Appendix S1** Supporting Information

**Table S1** Supporting Information

## Data Availability

The data that supports the findings of this study are available in the supplementary material of this article.
