# Erratum

**DOI:** 10.1002/ece3.9421

**Published:** 2022-10-24

In the recent article by Wu et al. (2022), the following affiliation for Minjun Dai was omitted in error:

“Central South University of Forestry and Technology, Hunan, China.”

And for clarity, the duplicate in‐text citations for “Liu, Feng, et al., 2019”, “Li, Zhang, & Griffith, 2021”, “Li, Zhang, Zhu, et al., 2021”, “Liu, Wang, et al., 2019” have been removed to avoid confusion.

These errors have been corrected in the online version.

We apologize for these errors.
