# Supplementary material for: Response of distribution patterns of two closely related species in Taxus genus to climate change since last inter‐glacial
Source: Ecol Evol. 2022 Sep 14;12(9):e9302. doi: 10.1002/ece3.9302 (PMC9475124; doi:10.1002/ece3.9302)
Supplement: Supplementary file 1 — Appendix S1 Supporting Information [file ECE3-12-e9302-s002.docx]

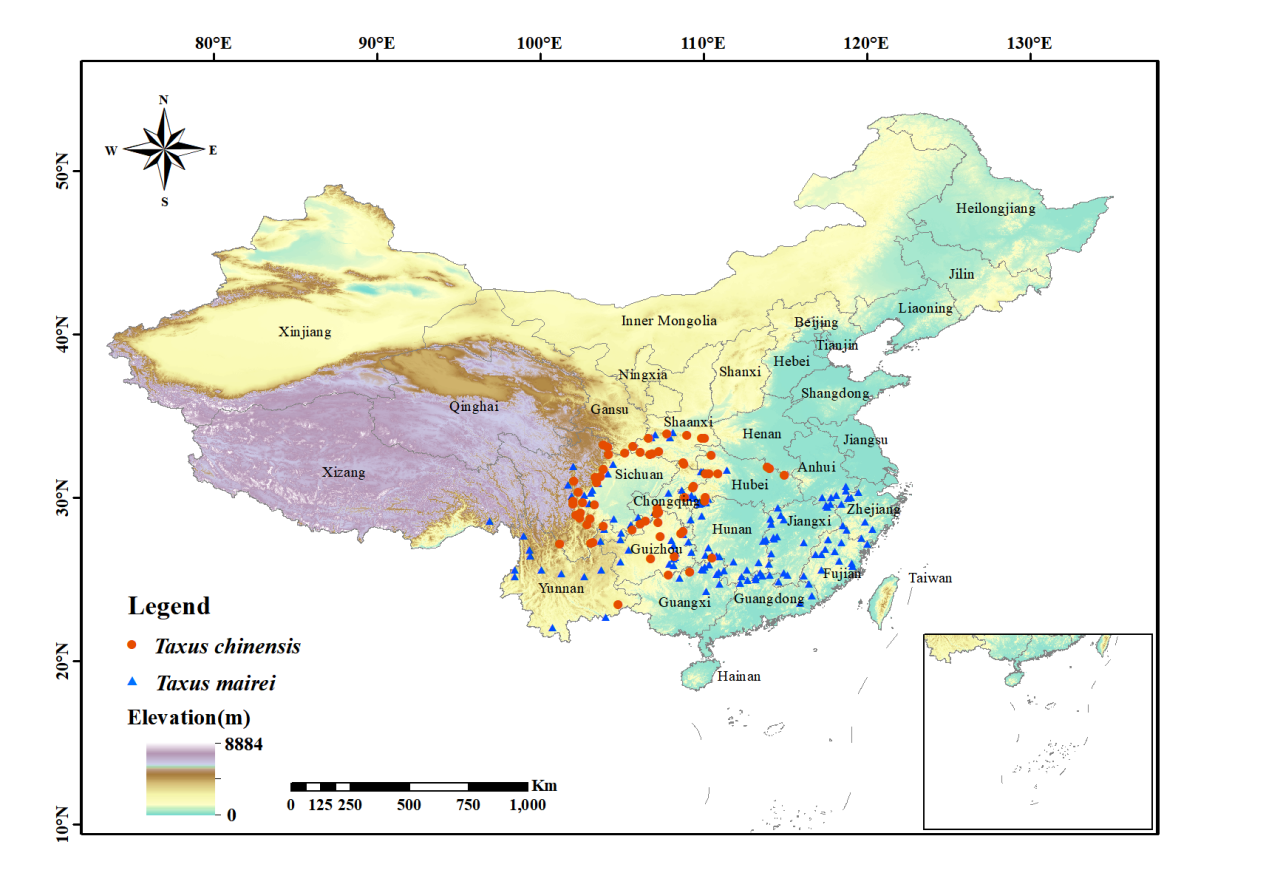


**FIGURE S1** Species occurrences points in current study


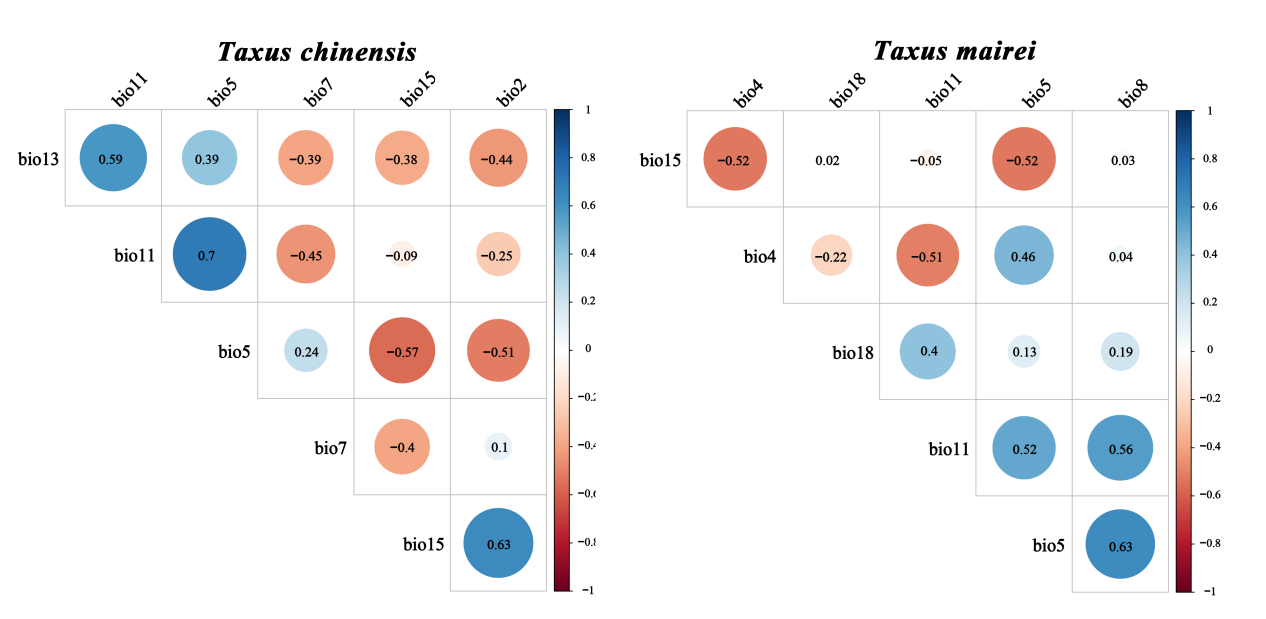


**FIGURE S2** Correlation matrix of six bioclim variables for the two *Taxus* species


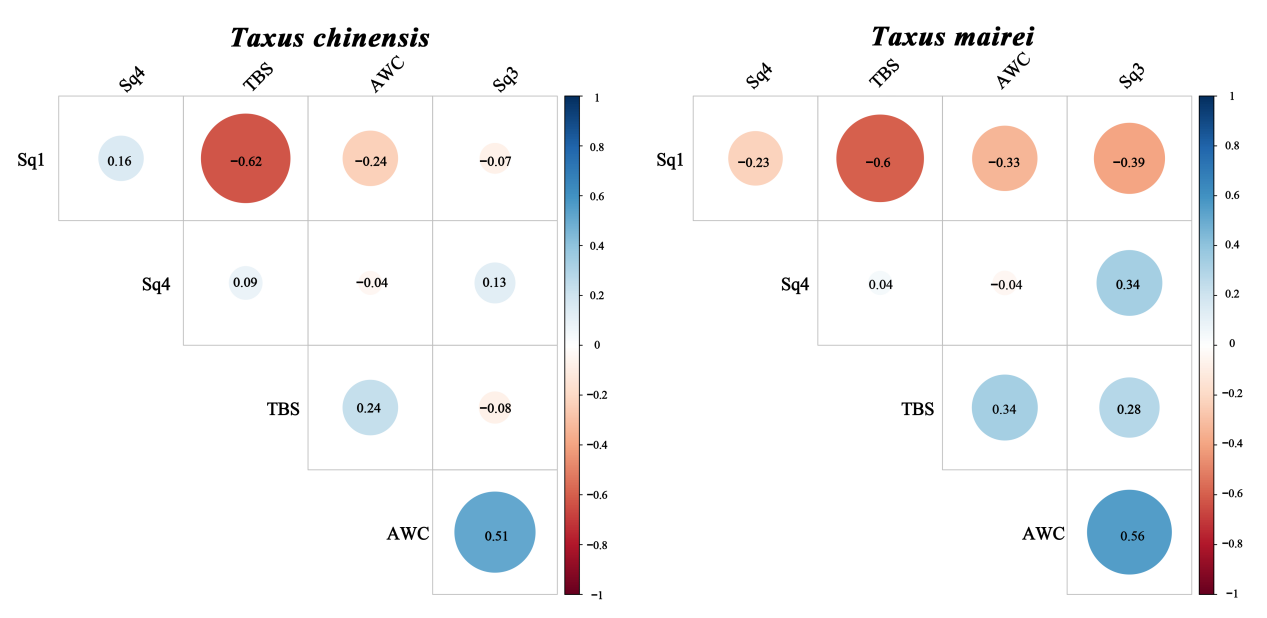


**FIGURE S3** Correlation matrix of five soil variables for the two *Taxus* species.

*Notes*: Sq1:Nutrient availability; Sq3: Rooting conditions; Sq4:Oxygen availability to roots; TBS:Topsoil base saturation; AWC: Available water storage capacity

*
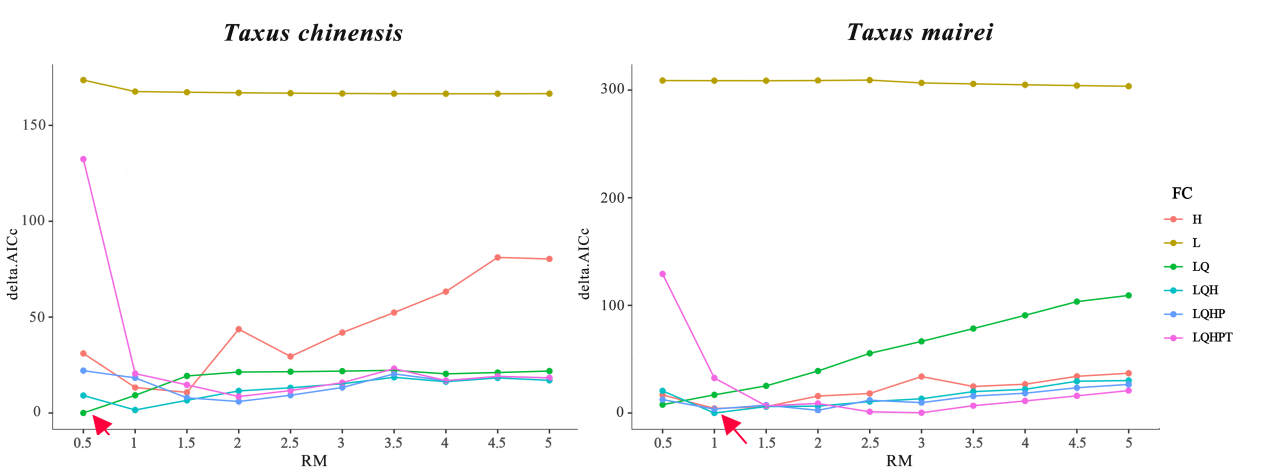
*

**FIGURE S4** Performances of species distribution models for two *Taxus* species under different parameter settings. Red arrows represent the optimized model parameters with the lowest AIC values (delta.AICc=0).


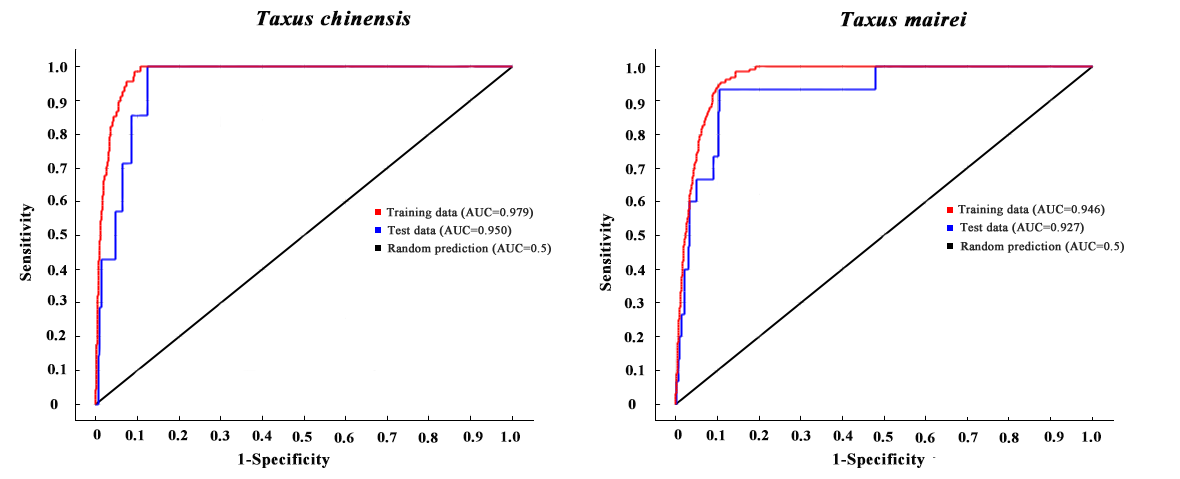


**FIGURE S5** Receiver operating characteristic (ROC) curve for *Taxus chinensis* and *Taxus mairei*

*
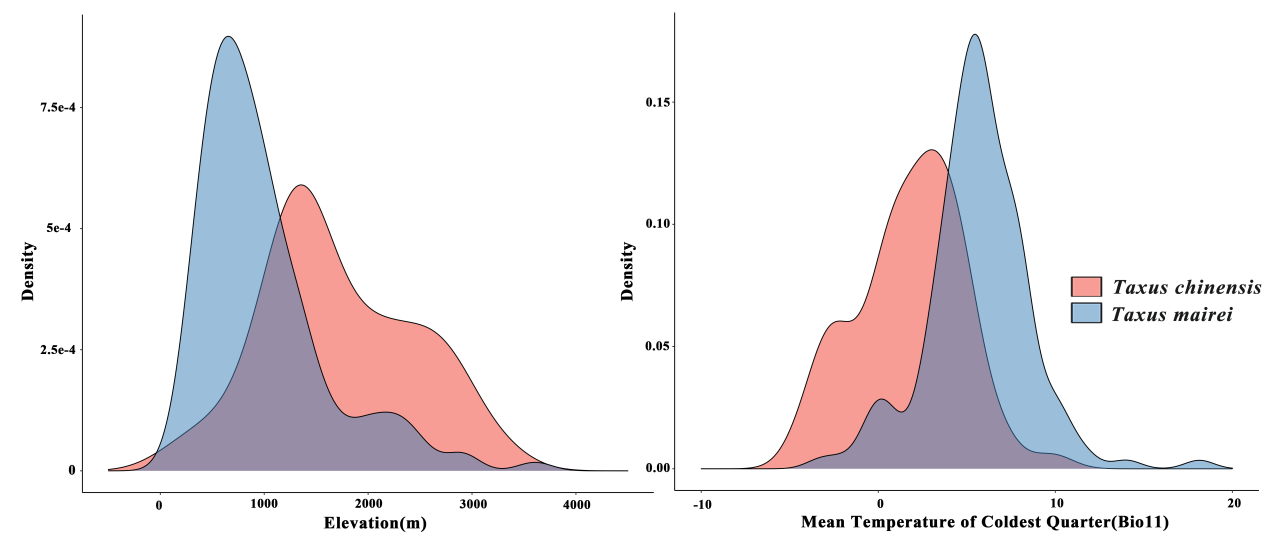
*

**FIGURE S6** Comparison of the *Taxus chinensis* and *Taxus mairei* response to bio11 and elevation at current period
